# Burnout among chiropractic practitioners: real or imagined an exploratory study protocol

**DOI:** 10.1186/2045-709X-20-4

**Published:** 2012-02-27

**Authors:** Shawn Williams, Stanley Innes

**Affiliations:** 1 Department of Health Professions, CUNY York College, 94 - 20 Guy R. Brewer Blvd, Jamaica, NY 11451, USA; 2 35 Maroondah Highway, Lilydale 3140, Australia

**Keywords:** Burnout, Chiropractic, Exploratory

## Abstract

Burnout is a psychological syndrome of emotional exhaustion, depersonalization and reduced personal accomplishment that has been found to exist in a significant number of healthcare and helping professionals. It imposes a significant societal burden by shortened practitioner lifespan, decreased efficiency, negative health outcomes and poorer levels of patient care. Theoretical models suggest that it appears to be the result of a complex interaction between job resources and job demands. It may be reasonable to conclude that Chiropractic professionals experience similar vocational demands and thus experience significant levels of occupational stress and subsequent burnout. However the data on burnout within the chiropractic profession is limited. It is possible that this results in significant negative outcomes on chiropractors and their patients. Therefore, the objective of this paper is to demonstrate the need to explore burnout in chiropractic practice and offer a research protocol for a potential study.

## Background

Past research has sought to explore and quantify the elevated levels of stress and susceptibility to burnout found in the helping professions. This has been demonstrated in professionals that share many similar characteristics to chiropractic such as medical practitioners [1,2], dentists [3-5], nurses [6-9], physical therapists [10-15], and occupational therapists [16-18]. However, this subject has never been studied in the chiropractic profession.

Factors that have been identified which increase levels of stress and susceptibility to burnout include constantly focusing on the needs of others, which may lead to fatigue, feelings of frustration and anger, a sense of ineffectiveness and failure, and the onset of depression and associated co-morbidities [2,19-22]. Other factors include physical workload, quality of recipient contact, physical environment, type of patient feedback, financial pressures and supervisor support [22]. These factors have been shown to vary across geographic locations [10-13,23].

With this body of evidence available for comparison it would seem logical to explore those significant factors already identified. Therefore, the objective of this paper is to demonstrate the need to explore burnout in chiropractic practice and to propose a research protocol for a potential study. Thus we would seek to question a representative selection of chiropractors from varying locations on their perceptions of the impact of these factors using an established and validated questionnaire.

### Literature review

In 2008 there were 49,100 registered chiropractors in the U.S.; of these approximately 44% were self-employed [24]. While there is a trend of increasing numbers of chiropractors there is counter pattern of declining use of chiropractic. In the U.S., 9.9% of U.S. adults reported having seen a chiropractor in 1997 vs. 7.4% in 2002. This was the largest relative decrease among CAM professions, which overall had a stable use rate [25].

Against this backdrop is the unfavourable public perception of chiropractic when compared with mainstream medicine with regard to ethics and honesty: in a 2006 Gallup Poll of U.S. adults, chiropractors were rated last among seven health care professions for being very high or high in honesty and ethical standards [26].

CareerCast.com rates chiropractic as the 10^th ^least stressful occupation in the USA in 2011 [27]. They based this on their perception that chiropractic involves low levels of physical demands, healthy offices, low levels of required stamina, low levels of competitiveness, no travel, few deadlines, no personal hazards, solid levels of income, and a positive outlook for employment and income growth. No significant sampling of chiropractors was undertaken in obtaining these results.

This perception would stand in contrast to a retrospective analysis [28] of licensed chiropractors in California. Here, alarmingly, the authors identified that the attrition in the first 10 years of practice rose from 10% to 25% in the past decade. The authors hypothesized that the rising attrition rate of chiropractors may be linked to a number of forces such as changes in population, an oversupply of chiropractors, changes in reimbursement, the cost of education, and chiropractors' general dissatisfaction with their own profession [28]. In addition, data from a recent pilot study [29] exploring the attitudes of non-practicing chiropractors suggest that business ethics, overhead expenses and salaries were contributing factors of attrition in the chiropractic profession.

A cursory glance on affiliated contemporary chiropractic material suggests that stress and burnout are salient and important issues. The first discussion on burnout was noted in 1987 in an ICA Review of Chiropractic [30]. Shelley Simon also wrote of this syndrome and its likely increasing prevalence in the Chiropractic newspaper Dynamic Chiropractic [31]. More recently it was raised in the American Chiropractors Association News [32] as an occupation whose practitioners are more prone to job burnout. A web search reveals a multitude of chiropractic associated organizations/assistants offering to help reduce stress levels by altering practice methods.

The authors contend that this milieu, and other factors, create high levels of vocational stress and may generate high levels of burnout. However there are mixed messages from the available information sources and to date there has been no research into this sizable and significant occupational group.

Although burnout and stress are separate concepts, it has been a commonly held belief that the cumulative effect of chronic stress often results in a condition known as burnout [33]. This has been particularly prevalent in the health care service industry [34]. If stress and burnout in the delivering of chiropractic care are at similar levels to that of other helping professions then accurate data, as opposed to anecdotal information, are required for a number of reasons.

First, burnout can negatively affect the chiropractic professional and in turn the quality of care delivered to the patient. Burnout has been implicated in poor patient outcomes in other caring professions [11,12]. It is, therefore, possible that higher burnout levels result in poorer clinical decisions and treatment applications with an increased incidence of adverse outcomes.

Second, Mertz et al. (2010) suggest that most non-practicing chiropractors believed that the practice of chiropractic medicine was not a good career choice and would not recommend someone choosing chiropractic as a vocation [29]. Research has suggested that chiropractic colleges rely on practicing chiropractors as major recruiters of students [35]. Increasing levels of burnout in the chiropractic profession may impact on this avenue of student recruitment for chiropractic colleges.

Third, this is a preventable syndrome [36,37]. Early recognition and intervention have been shown to reduce the negative impact on individuals, families, communities and health resources [36,37]. Similar actions may be needed in the chiropractic profession. Finally, the impact of stress and burnout on health care professionals may have significant consequences also on the provider [38]. On a personal level this has included a depletion of emotional and physical resources, an increased likelihood of feelings of incompetence, a lack of achievement (negative self-image) and neglect of self, coupled with family and broader societal obligations [39]. It has also been implicated in higher levels of mental illness, substance abuse, absenteeism, reduced productivity and compromised patient care [6,10,19,39-43].

The authors contend that with such potentially serious and long-term consequences, this issue can no longer be left unexplored in the chiropractic profession.

### Purpose and objectives of the study

The purpose of the intended study is to learn more about the levels of stress and burnout in the chiropractic profession. The specific objectives are to measure their prevalence and to compare those to levels previously identified and quantified in helping professions similar to the chiropractic profession.

## Methods/Design

### Study population and sampling

The authors intend to approach the appropriate chiropractic registration bodies with a request for assistance in distributing a mail out questionnaire to their membership. A pre-paid envelope with return address will be included. An alternative on-line survey option will also be made available.

For example, access to email listings of chiropractors is available for academic/educational purposes via a request through the State Board of Chiropractic Examiners, NJ. The investigators could request the email contact information of a randomized sample of licensed chiropractors from the State of New Jersey, State Board of Chiropractic Examiners, thus ensuring a systematic random sampling of the population of interest.

Subjects will include any Doctor of Chiropractic currently licensed to practice chiropractic and whom is at least 21 years old; with no limit on maximum age criteria.

Assuming a 60% return rate, the investigators will request the email contact information of a randomized sample of at least 500 licensed chiropractors in order to obtain sufficient numbers for the appropriate statistical analysis to be performed.

### Variables of interest and their rationale

While the term "burnout" has become quite common in both research and practice, it has been ill defined historically. Initially, the term burnout was introduced by Freudenberger in the 1970's, and was used as an informal expression by lawyers, social workers, psychiatrists, teachers and hospice counsellors, to denote their gradual energy depletion and loss of motivation and commitment that was often associated with a wide array of other physical and mental symptoms [41,44].

Maslach's (1982) definition of burnout is now the most influential and widely referenced [33]. It is described in their self-titled inventory (Maslach Burnout Inventory- MBI) as a psychological syndrome of emotional exhaustion (EE), depersonalization (DP), and reduced personal accomplishment (PA) that can occur among people who work with other people in some helping capacity. Here emotional exhaustion is seen to reduce a workers ability to provide support at a psychological level [45]. Depersonalization results in a negative and/or cynical attitude towards his or her client and/or work related responsibilities. The third aspect of burnout, reduced personal accomplishment, refers to the tendency to evaluate oneself negatively, such that one is no longer effective in working with clients and in fulfilling one's job responsibilities [2,45]. Their inventory has been shown to be both a reliable and valid tool in evaluating job burnout [2]. To date, the primary form of this instrument used to measure burnout among helping professionals is the MBI-HSS [2,11,38,44,46]. Three dimensions EE, DP and PA are rated on a 7-point likert scale (ranging from 0, "never" to 6, "every day"). Scores on each subscale are computed by summing the numeric responses. Permission to use the MBI-HSS will be obtained from Consulting Psychologists Press http://www.mindgarden.com/products/mbi.htm.

Respondents will also be asked to complete a second questionnaire that contains demographic questions (including gender, age, income levels and absenteeism) and open-ended questions related to chiropractic-specific stressors, as previously found in the literature. Examples of these include issues such as poor public perception, professional-identity inconsistencies, philosophical dissonance, negative media coverage, workplace injury levels from unique physical demands, market competition from other manual therapists, student loan default rates in North America and threats to autonomy [26,47,48].

### Data collection and management

An email letter of solicitation will be send to the random sample of emails via a group email distribution. This letter of solicitation will be in the body of the email and contain a link to an online version of the Maslach Burnout Inventory-Human Services Survey (MBI-HSS) and a brief demographic questionnaire developed by the primary investigator. SURVEY MONKEY (Gold member) software will be the electronic medium used. The Consent to Participate page shall include the title and purpose of the project, procedures for the anonymous and voluntary nature of the survey and potential risks and benefits to participants. The participants will complete the surveys in a place of their choice (e.g., their office, home or place of work), which has access to a computer. The Principal Investigator (PI) will conduct his portion of the research at York College, 94-20 Guy R. Brewer Blvd, Jamaica, NY 11451.

All survey related data will be stored on a thumb drive, which will be stored in locked cabinet at the place of research. After a minimum of 3 years, the PI will destroy the information on the thumb drive.

### Analysis of data

This study is conducted with an observational research design that will involve the use cross-sectional data collection as a means of establishing the prevalence of burnout among chiropractors and to examine its association with some potential risk factors.

The primary analysis will involve descriptive summary statistics (means, standard deviations) and inferential statistics (confidence intervals) for estimating the prevalence of burnout. The use of univariate analysis using t-tests and chi-square statistic will be utilized to compare the rate of burnout with the factors under investigation. Chiropractic-specific stressors will be correlated using Pearson correlation. Moreover, the relationship between the dimensions of burnout and ratio-level demographic variables will be tested using bivariate Pearson correlational analyses. Spearman's rho analyses will we used for ordinal-level data. To control for possible confounding factors, a multiple logistic regression will be utilized with burnout as the dependent variable.

### Ethical considerations

Research ethics and ethics approval will be sought from the principal investigators academic institution at the School of Health & Behavioral Sciences, CUNY - York College, New York, United States of America. All responses will be kept completely confidential and investigator(s) will be blinded to response and subject identity connectors.

## Discussion

### Summary of proposed study, need for the study and benefits of the study

Chiropractors are significant members of the health care community. Past studies have shown that the health care profession population experiences high levels of stress and burnout. Previous studies have identified ameliorable factors, which can result in the implementation of effective interventions with outcomes that include improved levels of patient care and practitioner health measures. No such exploration has been offered in the chiropractic profession; consequently an evidence-based intervention is not possible. The authors contend that now is the time.

## Abbreviations

U.S.: United States: EE: Emotional exhaustion; DP: Depersonalization; PA: Personal accomplishment; PI: Principle investigator.

## Competing interests

The authors declare that they have no competing interests.

## Authors' contributions

SW is the PI of the proposed study, and the original proposal author. SI added content expertise and editorial modifications. Both authors read and approve the final version of the manuscript.
